# Acute Alpha-Glycerylphosphorylcholine Supplementation Enhances Cognitive Performance in Healthy Men

**DOI:** 10.3390/nu16234240

**Published:** 2024-12-09

**Authors:** Chad M. Kerksick

**Affiliations:** Exercise and Performance Nutrition Laboratory, Department of Kinesiology, Lindenwood University, St. Charles, MO 63301, USA; ckerksick@lindenwood.edu; Tel.: +1-636-627-4629

**Keywords:** cognition, nootropic, mental, output, mental acuity

## Abstract

Background: Choline is an essential nutrient required for proper cell functioning. Due to its status as a precursor to acetylcholine, an important neurotransmitter connected to cognition and neuromuscular function, maintaining or enhancing choline levels is of interest. Supplementation with alpha-glycerylphosphorycholine (A-GPC) can maintain choline levels, but its ability to offer support towards cognition remains an area of ongoing research. Methods: Using a randomized, double-blind, placebo-controlled, crossover approach, 20 resistance-trained males (31.3 ± 11.0 years, 178.6 ± 7.3 cm, 84.6 ± 11.4 kg, 15.4 ± 5.6% body fat) consumed either a placebo (PL), 630 mg A-GPC (HD), or 315 mg (LD) A-GPC (GeniusPure^®^, NNB Nutrition, Nanjing, China). After resting hemodynamic assessments, participants took their assigned dose and had cognitive assessments (Stroop, N-Back, and Flanker), visual analog scales, and hemodynamics evaluated 60 min after ingestion. All participants then warmed up and completed vertical jumps and bench press throws before completing a bout of lower-body resistance exercise (6 × 10 repetitions using the Smith squat at a load of 70% 1RM). Venous blood was collected 5, 15, 30, and 60 min after completion of the squat protocol to evaluate changes in growth hormones, and follow-up visual analog scales and cognitive measurements were evaluated 30 min after completing the exercise bout. Results: When compared to PL, changes in Stroop total score were statistically greater after HD (13.0 ± 8.2 vs. 5.2 ± 9.0, p = 0.013, d = 0.61) and LD (10.8 ± 7.7 vs. 5.2 ± 9.0, *p* = 0.046, d = 0.48) administration, in addition to significantly faster times to complete the Stroop test in the HD group when compared to PL (−0.12 ± 0.09 s vs. −0.05 ± 0.09 s, *p* = 0.021, d = 0.56). No significant differences between groups were found for the Flanker and N-Back assessments, while a tendency was observed for HD to have faster reaction times when compared to PL during the Flanker test. No group differences were realized for visual analog scales, physical performance, or growth hormone. Statistically significant changes in heart rate and blood pressure were observed in all groups, with all recorded values aligning with clinically accepted normative values. Conclusions: HD and LD A-GPC supplementation significantly increased cognitive performance in a group of young, healthy males as measured by changes in the Stroop Total Score and completion time of the Stroop test. These results offer unique insight into the potential for A-GPC to acutely increase cognition in a group of young, healthy males. While previous research has indicated potential for A-GPC to acutely improve cognition in clinical populations, extending these outcomes to healthy individuals can be potentially meaningful for a wide variety of populations such as athletes, race car drivers, military operators, and other non-athletic populations who desire and have a need to improve their mental performance. This study was retrospectively registered as NCT06690619 on clinicaltrials.gov.

## 1. Introduction

Choline has established itself as an important nutrient essential for proper functioning of many organs in the human body [1]. As part of its status as a critical component of the neuronal phospholipid bilayer, choline is a fundamental part of signal transduction and methylation of DNA, histones, and neurons. Choline is a precursor of acetylcholine (ACh), a neurotransmitter involved in memory, attention, and skeletal muscle contraction [1]. As such, ACh is a key component of communication throughout the nervous system, thus creating its theorized connection to cognition, while ACh also sits at the coalface between the nervous and muscular systems at the neuromuscular junction. Previous work has indicated that supplementation with alpha-glycerylphosphorylcholine (A-GPC), a choline-containing phospholipid, can increase choline levels, and in doing so may augment cognition, physical performance, and endogenous hormone production [2,3,4].

Due to A-GPC’s ability to increase choline levels [5], as well as other evidence that demonstrates disruption in cholinergic transmission leading to learning and memory deficits in times of reduced ACh availability [6], the interest in A-GPC supplementation to support cognition has been evident for several years. Currently, research findings are mixed regarding A-GPC’s potential to impact cognition [7,8]. When investigated in clinically compromised or healthy aged populations, A-GPC studies have indicated that A-GPC supplementation enhances memory and cognitive function and is an effective treatment for neurodegenerative disorders such as Alzheimer’s disease and dementia [1,9]. A-GPC has also been proposed to offer treatment support for traumatic brain and other cerebrovascular accidents [10]. Research in healthy populations, however, is scant, and, to date, the majority of these studies have failed to highlight cognitive benefits in healthy individuals [11,12]. One of the initial studies exploring A-GPC for its ability to impact cognition used 32 healthy young volunteers who were administered an intramuscular injection of 1000 mg A-GPC or placebo for ten days before having their cognition evaluated [13]. The authors reported that A-GPC administration was able to antagonize memory and attention impairment induced by scopolamine. Unfortunately, the method of administration (intramuscular injection) in this study challenges its translation to larger segments of the population. A 2021 study by Tamura et al. [4] administered daily 400 mg doses of A-GPC or a placebo for 14 days to healthy volunteers who completed a scale to evaluate emotional states. After the treatment was administered, motivation levels were significantly improved, while anxiety levels were not impacted. Two other studies in healthy individuals examined the potential for A-GPC to impact cognition. The first study was completed by Parker et al. [12], who used a randomized, double-blind, placebo-controlled, crossover design and reported that the performance in the serial subtraction test for the 200 mg dose of A-GPC scores was 18.1% and 10.5% faster than the caffeine and placebo groups, respectively, in 20 healthy men and women. There were 20 healthy men and women who completed visual analog scales, a serial subtraction test, and a battery of physical performance tests after ingesting either 200 mg of A-GPC, 200 mg of caffeine, 400 mg of A-GPC, or a placebo. No statistically significant changes were observed in the serial subtraction test, although the scores for the 200 mg dose of A-GPC group were 18.1% and 10.5% faster than the caffeine and placebo groups, respectively. Additionally, Bunn and colleagues [11] acutely supplemented 21 college-aged males in a randomized, crossover manner to receive either a placebo or a combination of 500 mg A-GPC, 250 mg of uridine-5′-monophosphate, and 1500 mg DHA. During each condition, participants had their cognition assessed using the ImPACT protocol along with vertical jump and maximal bench press repetitions. The results from this study also indicated that a single day of supplementation exerted no impact on their outcomes.

Other studies have also explored the potential for A-GPC to positively influence various indicators of muscular performance [3,5,12,14]. The results from these studies are currently mixed, which may be due to the length and amount of supplementation provided in each study. Three peer-reviewed studies are available that support the ability of A-GPC to augment exercise performance. Bellar et al. [3] supplemented 13 young, healthy males with either 600 mg A-GPC or placebo for six days and reported significant improvements in lower body force production. Another study involving young, healthy males required the participants to supplement with either a placebo, 250 mg A-GPC, or 500 mg A-GPC in a randomized, double-blind fashion for seven consecutive days [5]. Serum choline was increased, along with significantly greater maximum velocity and maximum power values during a countermovement jump. Finally, Harrington et al. [15] reported that muscular power was improved in 30 male trained cyclists after seven days of supplementing with 300 mg of A-GPC, but the A-GPC was provided as part of a combination that also included BCAAs and citrulline, so the independent impact of A-GPC was not able to be clearly discerned from this study.

Three reports are available that outline findings after a single dose was delivered. In an abstract presented as part of conference proceedings, Ziegenfuss et al. [14] used a randomized, double-blind, placebo-controlled, crossover approach with seven healthy men to examine the impact of consuming a single 600 mg dose of A-GPC 90 min prior to completing a bout of lower-body resistance exercise and reported that the peak force production (*p* < 0.02) during a series of bench press throws was greater when A-GPC was provided, while no changes in peak velocity or peak power were observed. Alternatively, no changes in vertical jump performance were reported in an abstract presented as part of conference proceedings by Parker et al. [12], who supplemented 20 healthy men and women with either 200 mg of A-GPC, 200 mg of caffeine, 400 mg of A-GPC, or a placebo. Finally, Bunn and colleagues [11] acutely supplemented 21 college-aged males in a randomized, crossover manner to receive either a placebo or a combination of 500 mg A-GPC, 250 mg of uridine-5′-monophosphate, and 1500 mg DHA and reported that supplementation did not impact vertical jump performance or the maximal number of bench press repetitions completed.

A final area of interest has been in the ability of A-GPC to heighten endogenous production of growth hormone secondary to activation of the hypothalamic–pituitary axis. Kawamura et al. [2] supplemented eight healthy males with either 1000 mg A-GPC or a placebo in a randomized, double-blind, crossover fashion and reported that growth hormone levels were significantly increased 60 min after taking A-GPC. Another study was completed by Maldonado et al. [16] as part of a graduate thesis and has not been published in a peer-reviewed journal. It required overweight adults to supplement with 1200 mg of A-GPC each day for eight weeks. No changes in growth hormone were identified in this study. Finally, Ziegenfuss et al. [14] reported a significantly greater area under the curve for growth hormone in response to a heavy bout of lower-body resistance exercise when a 600 mg dose of A-GPC was provided when compared to placebo. With the popularity of A-GPC being used in an acute fashion as part of various nutritional formulations ingested prior to workouts and competitions, more research is needed to examine the ability of A-GPC to impact cognition and exercise performance. Therefore, the purpose of this study was to evaluate the impact of two different doses of A-GPC on cognitive and physical performance in healthy, resistance-trained individuals.

## 2. Materials and Methods

### 2.1. Overview of Research Design

All study participant recruitment and data collection were completed using a randomized, double-blind, placebo-controlled, crossover study design at the Center for Applied Health Sciences, a contract research organization in Canfield, OH, USA (www.thecahs.com). This study was retrospectively registered as a clinical trial as NCT06690619 on www.clinicaltrials.gov. Twenty-one healthy men with at least two years of resistance training experience were recruited to participate in this study protocol. Each study participant was required to complete four study visits, whereby study visit 1 was a screening visit. Visit 1 required each participant to observe an overnight fast and have a medical history completed, including assessments of their height, weight, body mass index, resting heart rate, and resting blood pressure, in addition to providing a 24 h dietary recall of their typical dietary intake, routine blood work (i.e., CBC, CMP, lipid panel) to establish eligibility, and familiarization with the cognitive and exercise battery used in subsequent visits. The provided 24 h dietary recall was reviewed for completeness with a research team member, and a copy was provided back to the study participant to aid them in replicating their food and fluid intake prior to each subsequent study visit. Eligible participants were then scheduled for study visit 2 approximately 3–7 days after study visit 1. Prior to study visit 2 and each subsequent study visit, participants were instructed to replicate their food and fluid intake for 24 h prior to each study visit; observe an overnight fast of at least 8 h; refrain from exercise for 48 h; and avoid caffeine, alcohol, and any foods that may impact choline levels for 24 h prior to each visit. Upon arrival, participants had their body mass, heart rate, and blood pressure evaluated and confirmed their compliance with all pre-study procedures. Participants then donated their first venous blood sample and consumed their assigned test product in front of a research team member. Using a randomized, double-blind manner, study participants ingested, during separate study visits (visit 2–4), either a 315 mg dose (LD) of alpha-glycerophosphorylcholine (A-GPC) (delivered as 350 mg of 90% A-GPC, GeniusPure^®^, NNB Nutrition, Nanjing, China), a 630 mg dose (HD) of A-GPC (delivered as 700 mg of 90% A-GPC, GeniusPure^®^), or a resistant dextrin placebo.

**Figure 1:**
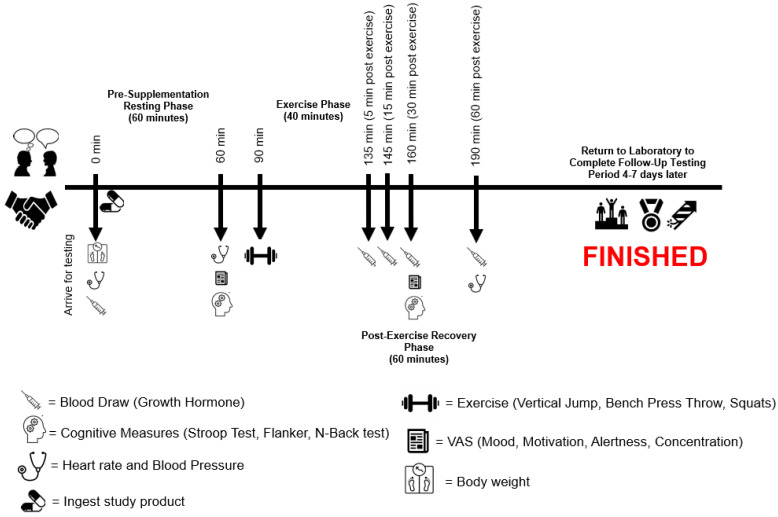
Timeline schematic of study design and procedures.

After supplement ingestion, participants then rested quietly for 60 min before having their resting heart rate and blood pressure reassessed. From there, visual analog scales for mood, motivation to perform physical and mental tasks, alertness, and concentration were completed, along with three assessments of cognitive function (Stroop, Flanker, and N-Back tests). Participants then completed a standardized warm-up and completed a series of body weight vertical jumps and bench press throws at 50% 1RM to evaluate lower-body and upper-body force, power, and velocity. Upon completion of the performance tests, a bout of lower-body resistance exercise (6 × 10 reps Smith Machine squats at 70% 1RM with 90 s of rest between each set) was performed, which was followed with venous blood collection 5, 15, 30, and 60 min after completion of the lower-body resistance exercise protocol to evaluate changes in growth hormone. An additional battery of visual analog tests and cognitive function tests was completed 30 min after completion of the lower-body exercise protocol. The final heart rate and blood pressure were assessed 60 min after completion of the exercise bout, and this completed the study visit. Study participants were then scheduled to return for their two remaining study visits (identical to study visit 2) approximately 4–7 days after their previous study visit (see Figure 1 and Table 1).

### 2.2. Study Participants

**Figure 2:**
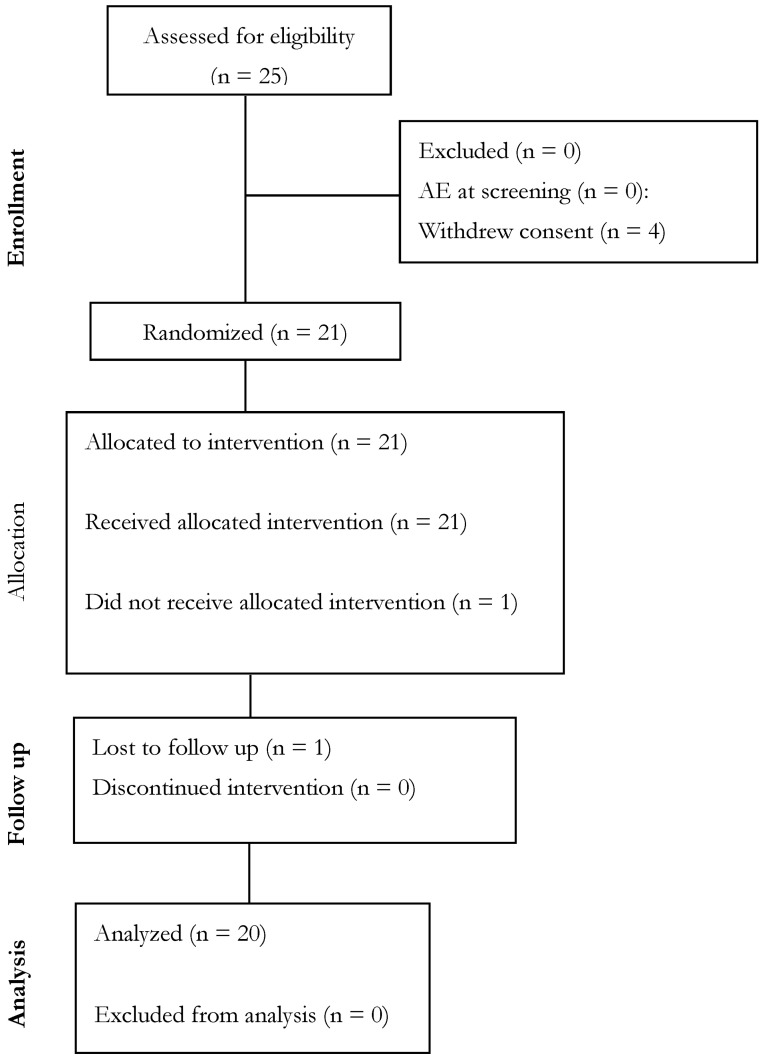
Consolidated Standards of Reporting Trials (CONSORT) diagram.

Twenty-one healthy, resistance-trained men between the ages of 20 and 55 years were recruited for this study from a local suburban community in Ohio (Figure 2). A priori sample size calculation was not completed, as the design for this study was modeled after Ziegenfuss et al. [14] which had a sample size of 7, and other studies (Parker et al. [12] and Bunn et al. [11]), which each had 20 participants and examined similar outcomes using a similar study design. Post hoc sample size evaluation indicated that an effect size of d = 0.5 with three groups, an alpha (α) level of 0.05, and an assumed power (1-β) of 0.80 would require a size of *n* = 14–15 per group. The complete demographics of all study participants can be found in Table 2. All participants read and signed an IRB-approved informed consent form prior to participating in the study (WCG IRB, Puyallup, WA, USA, Protocol # NNB-001-012024, Approval date: 17 January 2024). To be deemed eligible, all study participants were required to be in good health as determined by review of their medical history and routine blood chemistries by the research team. Inclusion criteria indicated that all participants were between the ages of 20 and 55 years and had body mass index levels between 18.5 and 34.9 kg/m^2^ (inclusive). Participants were required to replicate their previous 24 h dietary intake; avoid exercise for 48 h; refrain from alcohol, caffeine, and any foods or nutrients that may impact choline status for 24 h; and not make any changes to their medications, diet, or supplementation during the study protocol. Finally, participants were instructed to fast for eight hours prior to each visit.

Participants were excluded from participating if they indicated they were currently a competitive athlete or if they had a history of psychiatric, hepatorenal, musculoskeletal, autoimmune, neurologic diseases or disorders, as well as diabetes, asthma, gout, fibromyalgia, a clinical diagnosis of IBS/IBD, or a malignancy in the previous five years except for non-melanoma skin cancer. Participants with a prior history of gastrointestinal bypass surgery or chronic inflammatory condition or disease (e.g., rheumatoid arthritis, Crohn’s disease, ulcerative colitis, Lupus, HIV/AIDS, gastrointestinal or metabolic diseases that might impact nutrient absorption (e.g., short bowel syndrome, diarrheal illnesses, history of colon resection, gastro paresis, and inborn errors of metabolism)) were excluded. Alcohol consumption (>2 standard alcoholic drinks per day or more than 10 drinks per week), current smoker, clinically significant abnormal laboratory results, concomitant use of corticosteroids, or testosterone replacement therapy all resulted in study exclusion. Finally, participants who reported any current use of dietary supplements were required to maintain their current use throughout the study protocol. Individuals who were prescribed medications for anxiety or ADHD, who had a current allergy or sensitivity to any ingredient in the test formulation, or who had participated in another clinical research trial in the previous 30 days were excluded.

### 2.3. Procedures

#### 2.3.1. Anthropometrics

Using previously published methods [17,18], standing height was determined using a wall-mounted stadiometer and evaluated at study screening. Body mass was evaluated (±0.5 kg) using a Seca 767TM Medical Scale (Hamburg, Germany) and measured at each visit. Seated resting heart rate and blood pressure were measured after resting quietly for approximately five minutes using an automated blood pressure cuff (Omron HEM-780, Kyoto, Japan) at screening and again during each study visit approximately one hour and 3.5 h after supplement ingestion.

#### 2.3.2. Body Composition

Body composition was assessed for descriptive purposes using an InBody 570 (InBody Co., Ltd., Cerritos, CA, USA) bioelectrical impedance analyzer following an 8 h overnight fast for descriptive purposes. Prior to each analysis, participants voided their bladder; washed their hands; and removed any additional clothing, all metal jewelry, and any other accessories before wiping both hands and both feet clean with cleansing wipes (InBody Tissue; InBody Co., Ltd., Cerritos, CA, USA) provided by the manufacturer before standing erect with both hands and feet in contact with the sensors. On each day the machine was used, the device was calibrated according to manufacturer guidelines and device specifications.

#### 2.3.3. Dietary Intake and Physical Activity Monitoring

Outside of consuming their assigned supplementation dose at the beginning of each study visit, no other changes in dietary habits were prescribed as part of this study investigation. During study visit 1, all participants were instructed to complete a 24 h dietary recall. This recall was reviewed with research team members for accuracy and omissions. This food record was then copied, and participants were instructed to replicate their food and fluid intake the day prior to study visits 2–4. The collected dietary records were analyzed for average daily energy and macronutrient intake by trained study investigators (Nutritionix, Washington, DC, USA). During each study visit, participants were asked by research team members the extent to which they were able to follow a similar eating pattern as previous visits, and compliance was recorded. All study participants were required to refrain from exercise for 48 h prior to each study visit. Outside of those limitations, no other physical activity restrictions were required as part of this study investigation.

#### 2.3.4. Venous Blood Collection

A single blood draw was performed at visit 1 for screening purposes, and a series of five venous blood samples were collected at each study visit (visits 2–4). Whole blood and serum samples were collected using standard phlebotomy techniques at all study visits by a research nurse or phlebotomist. Whole blood samples were collected into K_2_-EDTA-treated tubes, and upon collection, each sample was slowly inverted ten consecutive times prior to immediate refrigeration. Serum samples were collected in serum separation tubes and allowed to clot for 30 min at room temperature prior to being centrifuged using a standard benchtop centrifuge for 15 min at 3200 rpm. Samples were analyzed by a commercial diagnostic laboratory (Lab Corp) for complete blood counts, comprehensive metabolic panels, lipid panels, and growth hormone.

#### 2.3.5. Supplementation Protocol

Using a Latin square to minimize order effects during this 3-way crossover, study participants were instructed to ingest either a resistant dextrin placebo, a low dose (LD: 315 mg) of A-GPC, or a high dose (HD: 630 mg) of A-GPC (GeniusPure^®^, 90% pure A-GPC, NNB Nutrition, Nanjing, China). All supplements were produced in non-transparent capsules that had similar colors, sizes, and shapes. All doses were administered by the research team at the beginning of each study visit and were consumed with approximately 12 fluid ounces of commercially bottled water. Third-party analysis confirmed the purity and content of A-GPC (batch # GP90-20230717, manufactured: 17 July 2023, re-test date: 16 July 2025, confirmed as 91.6%) and resistant dextrin (batch # 20230123, manufacture date: 23 January 2023, re-test date: 22 January 2025, confirmed 93.1%), as described.

#### 2.3.6. Visual Analog Scales

Visual analog scales (VAS) were completed 30 min prior to beginning the exercise protocol and 30 min after completing the exercise protocol to evaluate perceived changes in mood, motivation to perform physical and mental tasks, alertness, and concentration. Each VAS was 100 mm in length and anchored with “Worst Possible” or “Lowest Possible” and “Best Possible” or “Highest Possible”. These methods have been previously validated and determined to be reliable [19] and previously published [17,18,20,21].

#### 2.3.7. Cognitive Performance

The Stroop test is a widely used test to evaluate multiple parameters of cognitive performance, including attention, processing, cognitive flexibility, and the ability to inhibit cognitive interference [22], in addition to working memory [23]. The Stroop test requires individuals to read words printed in different-colored ink (for example, the word “green” could be printed in blue) and select the color of the ink they see instead of speaking the word they read [24]. All the participants were assessed using the congruent standard condition of the Stroop test with a duration of two minutes for each attempt/repetition. The outcome variables associated with the Stroop test were total score, accuracy, and average time per score.

The N-Back task is a widely used neurobehavioral assessment to evaluate working memory [25]. Briefly, this test requires participants to determine if the current stimulus repeats relative to the item that occurred ‘n’ times before its onset. The 1-back task was used in this study; thus, this task required participants to determine if the current stimulus was the same as the one before it. Stimuli were presented as a card with a colored shape for 1500 ms with a 500 ms intertrial interval. Participants had to respond as quickly as they could by striking an arrow on the keyboard corresponding to an appropriate response (e.g., either the color and shape matched, only the color or shape matched, or the color and shape did not match with the previous stimulus) [19,25]. An overall score, accuracy (correct responses as a percentage and number of correct responses out of the total number of attempts), total number of attempted responses, and reaction time (lumosity.com-Speed Match Overdrive) were used as part of analysis. Two separate tests were completed consecutively, with approximately 45 s between each test. The two tests were averaged to achieve one value for the score, accuracy (absolute and percentage), and reaction time per attempt, while the numbers of correct and attempted responses were totaled to provide a sum.

The Flanker Inhibitory Control and Attention Test measures attention and inhibitory control and assesses individuals’ ability to suppress responses that are inappropriate [26]. The test requires the participant to focus on a given stimulus while inhibiting attention to stimuli (shapes) flanking it. In doing so, the test evaluates speed and accuracy in responding to congruent stimuli, incongruent stimuli, and no stimuli. Scores are reported as accuracy and reaction time within each stimuli condition (congruent, incongruent, none).

During each study visit, two repetitions of each cognitive test were completed, and each test was administered approximately one hour and three hours after supplement ingestion. In total, each test was completed four times (eight times for the N-back) during each of the three separate study visits.

#### 2.3.8. Exercise Performance

Upper-body and lower-body performance were evaluated approximately 90 min after supplement ingestion, in alignment with previously completed data involving A-GPC supplementation [14]. Briefly, bench press throws were completed using a Tendo Power Analyzer (Lexington, SC, USA) unit attached to the end of a standard 20 kg barbell throughout each repetition to evaluate upper body peak power, average power, average velocity, and peak force. For each repetition, a submaximal load (i.e., 50% of perceived 1RM) was performed on a Smith machine. Subjects laid flat on their backs on a bench with their feet on the ground and hands on the bar in a pronated grip. Grip width was standardized for all subjects and reproduced during follow-up testing. The subjects lowered the bar (1–2 s eccentric action) until it lightly touched the chest slightly above the nipple line, and then explosively launched the bar vertically upwards. Using this approach, three sets of three repetitions with 90 s of rest between each set were completed, and the highest average and peak power, peak velocity, and peak force were recorded. Prior to each test, two normal bench press repetitions were allowed prior to the bench press throws as an orientation. The position of the Tendo analyzer was standardized to minimize any deviations of the lanyard and to minimize variability in the measurements. Previous studies have demonstrated the reliability of using a Tendo analyzer [27] for these outcomes, and similar methodological approaches have been published previously to evaluate the effects of various dietary supplements on these outcomes [28,29]. Lower body power was assessed via body weight jump squats while tethered to the Tendo power analyzer. A total of three countermovement jumps were completed. For each jump, subjects were required to hold a dowel on top of their shoulders and flex their knees eccentrically before executing the rapid vertical jump. Each jump was recorded for peak power and average power and peak velocity and force. The average of all three jumps was calculated and used for statistical analysis. Approximately 60–90 s of rest was given between sets. From these data, jump height was calculated [30,31] using algebraic substitution of the original formula: Jump height (cm) = average power in watts − (23 × body mass in kg) + (1393/21.2).

#### 2.3.9. Resistance Exercise Protocol

The resistance exercise protocol utilized in this study mirrored the approach utilized by Ziegenfuss et al. [14]. Herein and after completing all upper body and lower body performance assessments, study participants were guided through the completion of a lower-body resistance exercise protocol that was intended to stimulate endogenous hormone production [32,33]. The protocol commenced approximately two hours after consuming their assigned supplement and consisted of completing 6 sets of 10 repetitions at 70% 1RM using a squat movement on a Smith machine. A metronome was used (15 repetitions per minute) to ensure consistent cadence and time under tension. Approximately 90 s of rest was provided between each set of repetitions. A repetition was only counted if a full range of motion was utilized (thighs parallel to ground). All repetitions were supervised and counted by a study investigator/certified strength and conditioning specialist.

#### 2.3.10. Serum and Whole Blood Analysis

For screening purposes, blood collected during study visit 1 was analyzed for comprehensive metabolic panels (e.g., glucose, blood urea nitrogen [BUN], creatinine, aspartate aminotransaminase [AST], alanine aminotransaminase [ALT], lactate dehydrogenase, total bilirubin, alkaline phosphatase [ALP], glomerular filtrate rate, sodium, potassium, total protein, albumin, and globulin), total white blood cell and red blood cell counts, and lipid panels (e.g., triglycerides [TG], total cholesterol [TC], LDL cholesterol, HDL cholesterol). All analyses were completed using automated clinical chemistry analyzers (LabCorp, Dublin, OH, USA).

Growth hormone was measured in five venous blood samples across each supplementation period. In alignment with Figure 1 and to mimic the previous methods of Ziegenfuss et al., 2008 [14], growth hormone was assessed prior to supplementation and then 5, 15, 30, and 60 min after completing the lower-body resistance exercise protocol. The lower-body resistance exercise protocol did not begin until 60 min after supplementation ingestion, and the exercise protocol itself took approximately 40 min to complete. As such, post-exercise growth hormone levels ended up being assessed 135, 145, 160, and 190 min after ingestion of the supplementation, which corresponded with the post-exercise time points identified above in this section. Serum samples were processed as indicated and analyzed individually by LabCorp.

#### 2.3.11. Adverse Events

All adverse events (all local and systemic non-serious and serious) were monitored by the researchers and evaluated and assessed through reports coded using the Medical Dictionary for Regulatory Activities (MedDRA). In the event of an AE, the intensity of the AE was graded according to the protocol-defined criteria based on the Common Terminology Criteria for Adverse Events (CTCAE) Version 5.0, 2017.

#### 2.3.12. Statistical Analysis

A *p*-value of ≤0.05 was accepted as statistical significance, whereas *p*-values > 0.05 to ≤0.10 were accepted as a statistical trend. The normality of the frequency distributions was assessed using the Shapiro–Wilk test, and log transformations were applied when the data (or a majority of the data) suggested non-normality. All data provided in tables and figures display raw (non-transformed) values. A one-way repeated-measures ANOVA was used to assess differences between treatments for upper body performance, lower body performance, VAS variables, GH AUC, and change scores for cognitive testing. Change score/Delta values were computed by the differences in time points for the cognitive performance markers (30 min post-exercise–60 min post-supplement ingestion). Mixed factorial ANOVAs were used to evaluate the time and treatment main effects and time × treatment interaction effects for vital signs and growth hormone. When sphericity was violated, Greenhouse–Geiser adjustments were applied and LSD post hoc comparisons were used to evaluate pairwise comparisons. Any values >3SD or <−3SD were noted as outliers. Effect sizes using Cohen’s d (d) were calculated to evaluate the magnitude of the observed effect between groups (HD, LD, PL). A small effect size was ≥0.2, a medium effect ≥ 0.5, and a large effect ≥ 0.8. Analyses were conducted in GraphPad Prism, version 10.0.2.

## 3. Results

### 3.1. Compliance and Adverse Events (AEs)

Weekly compliance checks by the research study team revealed >95% compliance to the supplementation regimen. A summary table of adverse events (AEs) is provided (Table 3). Ten participants experienced AEs that were mild to moderate in nature during this study. There were no differences in proportions of reported adverse events (*p* > 0.05) between treatments. Given that all but one AE were experienced on visit 2 (i.e., the first testing visit) and proportional across study products, it was determined that most AEs were due to the study design procedures (e.g., IV insertions/blood draw and workout stress) rather than the study product consumed.

### 3.2. Body Mass and Hemodynamics

Body mass values tended to be different across the study protocol, with PL reporting significantly lower body mass values (84.4 ± 11.4 kg) than HD (85.0 ± 11.5 kg, *p* = 0.025, d = 0.54). Significant changes within groups occurred for hemodynamic variables. All measured outcomes remained within the clinically accepted values for healthy individuals (Table 4).

### 3.3. Cognitive Performance

**Figure 3:**
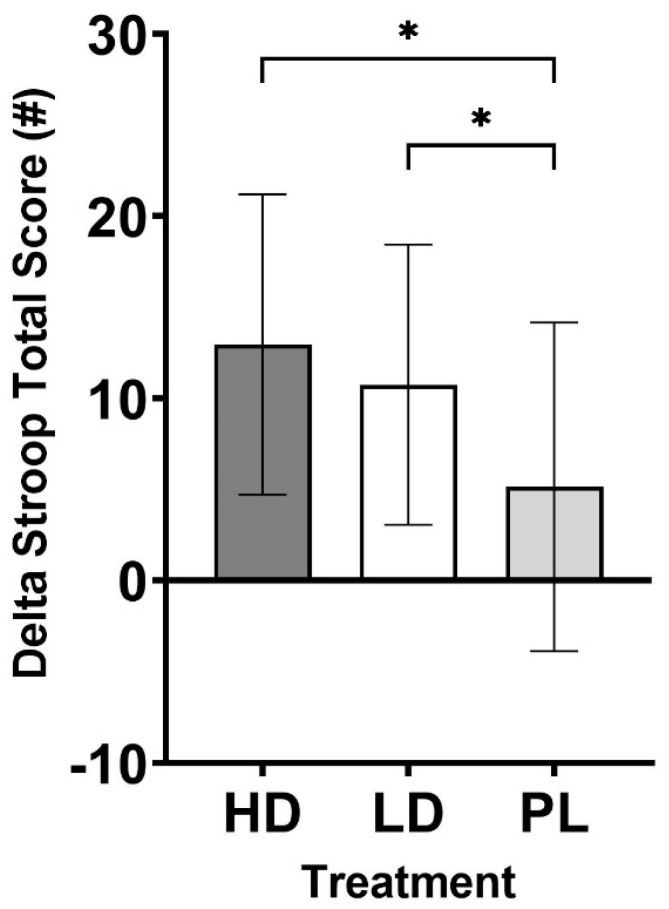
Delta Stroop total score. * = Different than PL (*p* < 0.05).

**Figure 4:**
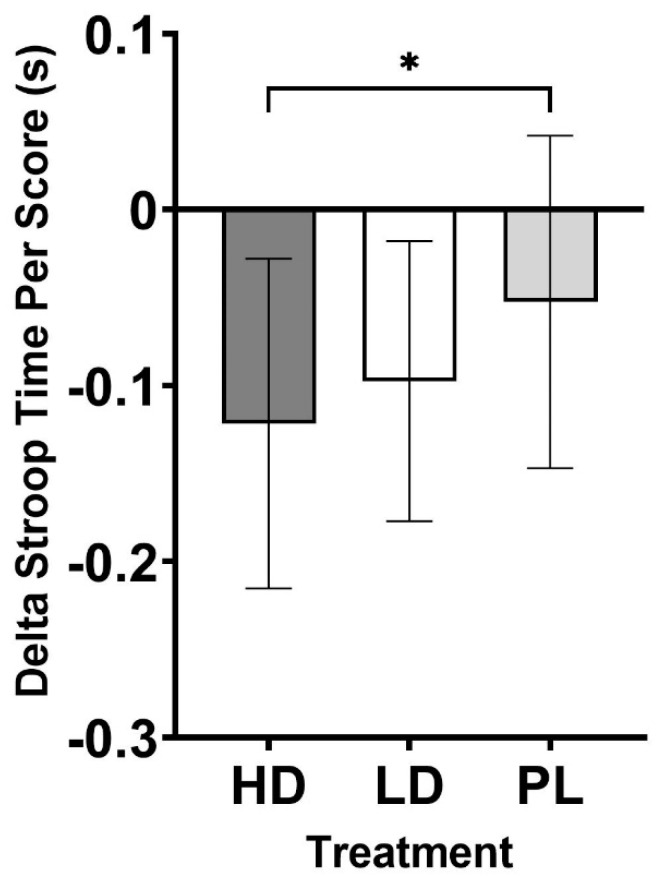
Delta time per score. * = Different than PL (*p* < 0.05).

Cognitive performance was evaluated using the Stroop color matching, Flanker, and N-back tests. Delta scores were computed to assess changes in Stroop performance and analyzed using one-way ANOVA. Using this approach, a statistically significant difference in Stroop total score (*p* = 0.016) was observed using one-way ANOVA. LSD post hoc comparisons revealed that the Stroop total score (Figure 3) increased to a statistically significantly greater degree in HD (13.0 ± 8.2; *p* = 0.013, d = 0.61) and LD (10.8 ± 7.7; *p* = 0.046, d = 0.48) when compared to PL (5.2 ± 9.0). A statistically significant difference was also observed with the time required per score in the Stroop test (*p* = 0.030; Figure 4). Following this, LSD post hoc comparisons revealed that HD resulted in significantly faster responses when compared to PL (HD: −0.12 ± 0.09 s vs. PL: −0.05 ± 0.09 s, *p* = 0.021, d = 0.56). A trend was observed for LD to be faster (−0.10 ± 0.08 s) than PL (*p* = 0.072, d = 0.42). Additionally, one-way ANOVA for Stroop accuracy indicated a statistical trend for differences (*p* = 0.073). LSD post hoc comparisons indicated that HD had significantly greater accuracy than LD (HD: 0.28 ± 0.85% vs. LD: −0.58 ± 0.90%, *p* = 0.005, d = 0.72). No statistically significant changes between groups (*p* > 0.05) were observed for measurements taken using the Flanker Compatible Accuracy (*p* = 0.376, Flanker Incompatible Accuracy (*p* = 0.754), Flanker Incompatible Reaction Time (*p* = 0.467), Flanker None Accuracy (*p* = 0.214), and N-Back tests (Table 5). Statistically different reaction times were observed for the Flanker Compatible Reaction Time, whereby HD exhibited statistically faster reaction times than PL (−51.2 ± 53.9 ms vs. −13.0 ± 51.6 ms, *p* = 0.018, d = 0.58).

### 3.4. Visual Analog Scales

As seen in Table 6, no significant differences between groups were identified 60 min after supplement ingestion for mood (*p* = 0.649), motivation to perform physical tasks (*p* =0.320), motivation to perform mental tasks (*p* = 0.664), alertness (*p* = 0.197), or concentration (*p* = 0.385). Compared to PLA, alertness at 60 min post-ingestion in the HD group tended to be greater (16.7%; *p* = 0.054, d = 0.47). When VAS was evaluated again 30 min after completion of the exercise bout, no significant differences were found between groups for mood (*p* = 0.649), motivation to perform physical tasks (*p* =0.320), motivation to perform mental tasks (*p* = 0.664), alertness (*p* = 0.197), or concentration (*p* = 0.385).

### 3.5. Physical Performance

Table 7 provides the data for the upper-body and lower-body performance tests. Using one-way ANOVA, no significant differences in bench press average power (*p* = 0.198), peak power (*p* = 0.168), peak velocity (0.296), or peak force (*p* = 0.159) were observed. To fully examine potential differences between the three groups, LSD comparisons were completed, and peak force production in HD was found to be significantly greater than PL (*p* = 0.043, d = 0.49). Peak force values tended to be greater in HD (*p* = 0.071, d = 0.43), and LD tended to be greater than PL (*p* = 0.073, d = 0.44).

Lower-body performance was evaluated using vertical jumps. Using one-way ANOVA, a statistically significant difference was observed for peak force produced (*p* = 0.050), with LSD post hoc comparisons indicating that LD was greater than HD (*p* = 0.027, d = 0.54) and LD tended to be greater than PL (*p* = 0.085, d = 0.41). Peak power (*p* = 0.085) tended to be different between groups, with forced LSD post hoc comparisons indicating that peak power values tended to be greater with LD than HD (*p* = 0.071, d = 0.43), and LD tended to be greater than PL (*p* = 0.068, d = 0.43). No differences were observed between groups using one-way ANOVA for average power (*p* = 0.954) and peak velocity (*p* = 0.160). Forced LSD post hoc comparisons indicated a tendency for HD to have greater peak velocity values when compared to PL (*p* = 0.091, d = 0.40).

### 3.6. Growth Hormone

As seen below in Table 8, the group × time interaction for growth hormone was not significant (*p* = 0.174), while the main effects of group (*p* < 0.001) and time were statistically significant (*p* < 0.001). The main effect of group was further decomposed by completing individual one-way ANOVA at each individual timepoint. No significant differences were observed between groups using one-way ANOVA for each individual timepoint (0 min: *p* = 0.366; 5 min: *p* = 0.069; 15 min: *p* = 0.362; 30 min: *p* = 0.318; 60 min: *p* = 0.447). Factorial ANOVAs with repeated measures on time were completed to evaluate changes within each group from the respective baseline values. In the HD and LD groups, observed growth hormone levels were increased at all post-exercise timepoints when compared to their respective baseline values. The PLA group was different at 5 min (*p* = 0.005) and 15 min (*p* = 0.013), but was not different from baseline 30 min (*p* = 0.158) and 60 min (*p* = 0.283) post-exercise. Area under the curve (AUC) calculations and one-way ANOVA indicated that no significant differences were found between the three groups (HD: 380 ± 279, LD: 414 ± 273, PL: 351 ± 272 ng/mL/min, *p* = 0.438).

## 4. Discussion

Choline availability can have a widespread physiological impact by way of impacting acetylcholine status across the body and facilitating other intracellular communication. A-GPC supplementation has been documented in studies to positively influence choline levels [5,34] and exert influence over mood [4], cognition [1,9,10], and physical performance [3,5]. The primary findings from the current study highlight the statistically significant improvement in Stroop total score when participants were supplemented with both HD and LD when compared to PL (Figure 3). Additionally, outcomes revealed that the HD group spent significantly less time (they completed the test faster) on the test when compared to PL (Figure 4). Forced post hoc tests also revealed that the HD group exhibited faster response times during the Flanker test when compared to PL (Table 5). Finally, although faster, HD exhibited decreased accuracy when compared to LD, but the accuracy was similar when compared to PL (Table 5). These results are meaningful because they represent, for the first time, that A-GPC supplementation is documented to increase parameters of cognitive performance in a young, healthy cohort. Previous investigations using this population were unable to identify such changes [11,12], while studies involving older and clinically compromised populations [1] have reported the ability of A-GPC to positively impact cognitive performance. The reasons for why these outcomes deviate from previous studies involving healthy populations are not entirely known. The current study and the two other investigations using single doses of oral ingestion both used robust randomized, double-blind, crossover, placebo-controlled study designs and made acute assessments of physical and cognitive performance. The Stroop color and matching test is a clinically validated and popular assessment of selective attention, cognitive flexibility, processing speed, and inhibition, while the other investigations used the ImPACT [11] and serial subtraction [12] tests. The higher A-GPC dosage (HD: 630 mg) during the present study was higher than the dosages provided during both the Parker (200 mg and 400 mg) and Bunn (500 mg) investigations [11,12], which may have impacted the outcomes, but the lower dose (LD: 315 mg) also exerted some positive influence, which somewhat rebuts this as an explanation for our outcomes. It is worth mentioning that the A-GPC delivered as part of the Bunn investigation was combined with two other ingredients (UMP and DHA), which further challenges one’s ability to compare the results of these investigations. Collectively, each of these studies used young, healthy cohorts (20–30 years) and completed measurements within a similar time frame of administration (30–90 min after ingestion). Another difference may be the manufacturing method utilized for the A-GPC provided in the current investigation versus the other investigations. The A-GPC used in the present study (GeniusPure^®^, NNB Nutrition) is commercially produced at a higher purity rate in a manner that is free of soy and other common allergens. The extent to which commercial production methods may have impacted the bioavailability or absorption kinetics is not currently known.

The present study also examined the potential impact of A-GPC’s ability to augment physical performance using a study approach that closely mimicked the study design previously utilized by Ziegenfuss et al. [14]. Using this approach, the peak force produced during the vertical jumps was different between groups, with post hoc comparisons indicating that LD was greater than HD (*p* = 0.027, d = 0.54) and tended to be greater than PL (*p* = 0.085, d = 0.41) (Table 7). A trend was observed for vertical jump peak power using one-way ANOVA (*p* = 0.085) and forced LSD post hoc tests, with LD exhibiting a tendency to be greater than PL (*p* = 0.068, d = 0.43) and HD (*p* = 0.071, d = 0.43). Changes in upper-body performance were similar between all three groups using one-way ANOVA (Table 7), but forced post hoc tests indicated that a statistically significant difference was present between HD and PL (*p* = 0.043, d = 0.49). These results align somewhat with previous studies examining physical performance changes in young, healthy cohorts [3,5,14]. Briefly, Bellar et al. [3] reported statistically significant improvements in peak force production, while Marcus et al. [5] reported greater peak power production when A-GPC was provided. In addition, and in alignment with the present study, Ziegenfuss et al. [14] measured a series of upper-body and lower-body outcomes and reported significant increases in upper-body force versus placebo and a statistical trend for A-GPC to be greater than PL. Both the Bellar and Marcus studies dosed orally for six and seven days, respectively, while Ziegenfuss delivered single oral doses similar to the present study. Thus, it seems plausible that, if a longer supplementation protocol had been followed in the present study, that force and power production may have been further augmented to yield even stronger outcomes related to performance. Future research should examine this as a possibility.

The present study also sought to examine changes in perception of affect (mood, motivation, etc.) and growth hormone changes in response to an intense, acute bout of lower-body resistance exercise. Using visual analog scales, perceptions of mood, alertness, concentration, and motivation towards physical and mental exercise were assessed, and no statistically significant changes were observed between any groups. The working hypothesis for these measurements suggested that the known increases in choline which occur with A-GPC supplementation may have improved these outcomes, particularly when surrounded by a challenging acute bout of exercise. Moreover, Tamura et al. [4] previously showed that 14 days of 400 mg of A-GPC given orally can positively impact mood, but no changes in these variables were observed in the present study. Thus, it seems prudent to suggest that future studies should examine whether longer dosing protocols would demonstrate changes in these outcomes. Circulating growth hormone levels were also evaluated after A-GPC supplementation, as it has been shown in previous studies to increase growth hormone levels [2,14,35]. In contrast to the results provided by Ziegenfuss et al. [14], no differences in growth hormone concentrations were observed in the present study. While our observed changes in growth hormone align with previous investigations that reported on growth hormone changes after acute bouts of resistance exercise [36,37], the lack of changes secondary to A-GPC delivery was somewhat unexpected considering the similar dosages used and the study design replication that was employed in the Ziegenfuss study [14]. A ceiling effect surrounding growth hormone response is evident in this literature, which could have limited the potential for A-GPC to further augment this response. Whether or not differences existed within the A-GPC remains to be seen, and future studies using multiple higher doses should be completed in order to continue to explore the ability of A-GPC to function as a growth hormone secretagogue.

The current paper has a few key strengths to highlight, starting with the randomized, double-blind, placebo-controlled, crossover study design used to examine the measured outcomes. In addition, the current study also provides insight into any dose–response outcomes which may occur with A-GPC supplementation. Other key strengths lie in the completion of both cognitive and physical performance outcomes after one dose of supplementation.

This paper also has limitations for the reader to consider that should be highlighted when evaluating the findings. We only recruited males into this study to best align our outcomes with the current literature, and as a result, more research involving females should be completed to establish how well these results hold true for females. In addition, many other populations (healthy, aged, sleep-deprived shift workers, race car drivers, military operators, pilots, clerical staff, competitive athletes, etc.) could potentially find these outcomes to be of interest. As such, findings from the present study are limited to healthy, physically active males who are not competitively active in some form of sports, and thus, more research should be explored in some of these other populations. A full discovery of pairwise comparisons using LSD approaches was completed for primary and secondary outcomes to clearly understand all potential differences. It is acknowledged and should be considered by the reader that using more rigorous correction for pairwise comparisons may have impacted our final outcomes. Another key limitation was the single dose delivered as part of this study design. While results after a single dose have obvious value, being able to understand how the findings from our current study design are impacted by longer supplementation regimens would also be of interest, particularly if longer doses are also considered.

## 5. Conclusions

In conclusion, a single 630 mg dose of A-GPC was shown from the present study to positively impact total Stroop test scores and processing speed and tended to impact some conditions of the Flanker test, while a lower dose demonstrated tendencies for improved performance when compared to placebo.

## Figures and Tables

**Table 1:** Overview of study design.

Procedure	Visit 1 (Screen)	Visit 2 (Day 1)	Visit 3 (Day 8)	Visit 4 (Day 15)
Informed Consent	X			
Inclusion/Exclusion Criteria	X			
Medical History	X			
Height, weight, and BMI	X	X	X	X
24 h Dietary Recall	X			
CBC, CMP, Lipid Panel	X			
Vitals (HR and BP)	X			
Body Composition	X			
Upper Body Exercise Performance		X	X	X
Lower Body Exercise Performance		X	X	X
Resistance Exercise Protocol		X	X	X
Cognitive performance	X	X	X	X
Growth hormone		X	X	X
Visual Analog Scales		X	X	X
Vitals (HR and BP) Post-Exercise		X	X	X
24 h Diet Records/Analysis/Repeat		X	X	X
Protocol Compliance		X	X	X
Dispense Test Product		X	X	X
Adverse Events Monitoring	X	X	X	X

**Table 2:** Study participant characteristics (*n* = 20).

Variable	Mean ± SD
Age (years)	31.3 ± 11.0
Height (cm)	178.6 ± 7.3
Weight (kg)	84.6 ± 11.4
Body Mass Index (kg/m^2^)	26.4 ± 2.5
Body Fat (%)	15.4 ± 5.6
Systolic Blood Pressure (mm Hg)	120.7 ± 12.1
Diastolic Blood Pressure (mm Hg)	72.3 ± 9.0
Resting Heart Rate (bpm)	63.4 ± 8.7
White Blood Cell Count (×10^3^/µL)	5.3 ± 1.4
Red Blood Cell Count (×10^6^/µL)	5.3 ± 0.4
Hemoglobin (g/dL)	15.8 ± 0.7
Hematocrit (%)	46.5 ± 2.1
Glucose (mg/dL)	90.0 ± 9.8
Blood Urea Nitrogen (mg/dL)	16.8 ± 5.3
Creatinine (mg/dL)	1.1 ± 0.2
BUN/Creatinine ratio	91.8 ± 16.9
eGFR (mL/min/1.73)	14.7 ± 3.5
Sodium (mmol/L)	140.8 ± 1.9
Potassium (mmol/L)	4.5 ± 0.4
Chloride (mmol/L)	103.1 ± 2.0
CO_2_ (mmol/L)	23.9 ± 1.5
Calcium (mg/dL)	9.5 ± 0.3
Total Protein (g/dL)	7.1 ± 0.4
Albumin (g/dL)	4.7 ± 0.2
Globulin (g/dL)	2.4 ± 0.3
Albumin/Globulin ratio	2.0 ± 0.3
Bilirubin (mg/dL)	0.6 ± 0.3
Alkaline Phosphatase (IU/L)	69.3 ± 14.4
AST (IU/L)	23.5 ± 5.7
ALT (IU/L)	25.7 ± 7.0
Total Chol (mg/dL)	162.0 ± 25.3
Triglycerides (mg/dL)	102.4 ± 112.1
HDL (mg/dL)	50.0 ± 10.6
VLDL (mg/dL)	18.6 ± 16.8
LDL (mg/dL)	93.4 ± 21.1
LDL/HDL	1.9 ± 0.4
Total/HDL	3.5 ± 1.0

**Table 3:** Summary of adverse events.

Treatment	HD	LD	PL
Severity			
Mild		2	
Moderate	2	2	4
Severe			
Relationship to Study Treatment (Product)			
Unlikely	1	1	1
Possible	1	3	3
Probable			
Relationship to Test Article (Procedural)			
Unlikely			
Possible			
Probable	2	4	4
Body Systems and AEs			
Gastrointestinal			
Emesis (regurgitate)		1	2
Nausea	1	1	1
Retching (dry heaving)	1		
Nervous			
Cephalalgia (headache)	1		
Cardiovascular			
Presyncope (lightheaded)		3	2
Total Number of Adverse Events Experienced During Study	3	5	5
Total Number of Subjects Experiencing AEs: *n* (%)	2/20 (10%)	4/21 (19%)	4/20 (20%)

**Table 4:** Hemodynamics.

		0 min Post-Ingestion	60 min Post-Ingestion	30 min Post-Exercise
Systolic Blood Pressure (mm Hg)	HD	121.1 ± 10.3	121.1 ± 11.4	116.4 ± 12.1
	LD	120.1 ± 9.3	120.4 ± 7.9	115.3 ± 11.6
	PL	123.1 ± 10.7	121.0 ± 9.8	116.9 ± 12.3
Diastolic Blood Pressure (mm Hg)	HD	74.9 ± 8.0	74.6 ± 7.4	73.5 ± 8.3
	LD	72.5 ± 8.6	77.5 ± 8.5 †#	71.9 ± 8.3 †
	PL	73.6 ± 7.2	76.8 ± 7.1	71.1 ± 7.0 †
Heart Rate (beats/min)	HD	62.6 ± 11.4	58.9 ± 10.2 †	72.6 ± 12.0 †
	LD	62.7 ± 8.7	60.7 ± 8.6	74.1 ± 8.6 †
	PL	61.3 ± 10.4	59.5 ± 9.7	74.2 ± 12.4 †

† = Different than 0 min post-ingestion (*p* < 0.05). # Different than HD (*p* < 0.05).

**Table 5:** Cognitive performance.

	HD	LD	PL	*p*
Stroop Total Score	13.0 ± 8.2 **	10.8 ± 7.7 **	5.2 ± 9.0	0.016
Stroop Accuracy	0.29 ± 0.85 ‡	−0.58 ± 0.90	−0.16 ± 1.35	0.073
Stroop Time Per Score (s)	−0.12 ± 0.09 **	−0.10 ± 0.08 *	−0.05 ± 0.09	0.030
Delta Flanker Compatible Accuracy (%)	−1.24 ± 3.1	−0.16 ± 2.57	0.00 ± 2.26	0.376
Delta Flanker Compatible Reaction Time (ms)	−51.2 ± 53.9 **	−13.0 ± 97.2	−13.0 ± 51.6	0.126
Delta Flanker Incompatible Accuracy (%)	−0.63 ± 2.97	0.00 ± 3.03	−0.78 ± 1.99	0.754
Delta Flanker Incompatible Reaction Time (ms)	−39.2 ± 60.1	−18.2 ± 107	−14.1 ± 48.8	0.467
Delta Flanker None Accuracy (%)	−1.25 ± 2.19	−0.39 ± 3.07	0.23 ± 0.76	0.214
Delta Flanker None Reaction Time (ms)	−34.1 ± 56.3 #	−11.5 ± 89.4	−19.4 ± 73.4	0.165
N-Back Score (au)	853 ± 1973	828 ± 1455	1486 ± 2328	0.490
N-Back Correct (#)	7.2 ± 7.5	5.2 ± 5.3	6.5 ± 7.0	0.617
N-Back Attempted (#)	9.2 ± 8.5	6.2 ± 5.4	7.5 ± 6.6	0.395
N-Back Accuracy (%)	−1.7 ± 2.5	−0.5 ± 2.2	−0.4 ± 3.0	0.241
N-Back Time Per Score (ms)	−81.7 ± 107	−71.7 ± 81.5	−67.4 ± 55.4	0.951

All values are reported and analyzed as delta scores (30 min post-exercise–60 min post-ingestion of assigned supplement). *p* = one-way ANOVA. * = Trend vs. PL (*p* ≤ 0.10). ** = Significantly different than PL (*p* ≤ 0.05). # = Trend vs. LD (*p* ≤ 0.10). ‡ = Significantly different than LD (*p* ≤ 0.05).

**Table 6:** Visual analog scales.

		60 min Post-Ingestion	*p*	30 min Post-Exercise	*p*
Mood	HD	7.2 ± 1.2	0.649	7.1 ± 1.3	0.189
	LD	7.3 ± 1.4		7.0 ± 1.7	
	PL	7.1 ± 1.3		6.5 ± 1.8	
Motivation Towards Physical Exercise	HD	6.6 ± 1.6	0.320	5.7 ± 2.4	0.546
	LD	6.9 ± 1.4		5.4 ± 2.5	
	PL	6.4 ± 1.4		5.1 ± 2.6	
Motivation Towards Mental Exercise	HD	6.8 ± 1.7	0.664	6.3 ± 1.9	0.182
	LD	6.9 ± 1.6		6.6 ± 2.1	
	PL	6.6 ± 1.7		5.7 ± 2.2	
Alertness	HD	7.2 ± 1.3	0.197	6.3 ± 2.2	0.860
	LD	6.7 ± 2.3		6.3 ± 2.6	
	PL	6.2 ± 2.5		6.1 ± 2.6	
Concentration	HD	7.2 ± 1.3	0.385	6.5 ± 1.7	0.942
	LD	7.2 ± 1.4		6.5 ± 2.1	
	PL	6.9± 1.5		6.6 ± 1.8	

*p* = *p*-value of one-way ANOVA.

**Table 7:** Physical performance.

	HD	LD	PL	*p*
Bench Press Average Power (watts)	509 ± 94	527 ± 107	506 ± 101	0.198
Bench Press Peak Power (watts)	913 ± 192 #	909 ± 213	864 ± 202	0.168
Bench Press Peak Velocity (m/s)	1.36 ± 0.22	1.38 ± 0.23	1.35 ± 0.23	0.296
Bench Press Peak Force (N)	898 ± 168 **	915 ± 210 *	869 ± 193	0.159
Vertical Jump Average Power (watts)	1572 ± 263	1569 ± 286	1573 ± 278	0.954
Vertical Jump Peak Power (watts)	7309 ± 2719 #	8403 ± 4363 *	7546 ± 3555	0.085
Vertical Jump Peak Velocity (m/s)	3.45 ± 0.44 *	3.42 ± 0.45	3.37 ± 0.45	0.160
Vertical Jump Peak Force (N)	2899 ± 1155 ‡	3080 ± 1060 *	2916 ± 1192	0.050

*p* = one-way ANOVA. * = Trend vs. PL (*p* ≤ 0.10). ** = Significantly different than PL (*p* ≤ 0.05). # = Trend vs. LD (*p* ≤ 0.10). ‡ = Significantly different than LD (*p* ≤ 0.05).

**Table 8:** Growth hormone (ng/mL).

Time	HD	LD	PL		*p*
0 min	0.58 ± 1.38	0.72 ± 2.12 †	0.35 ± 0.59 †	Group	<0.001
5 min	9.82 ± 6.72 †	14.00 ± 12.66 †	11.63 ± 11.46 †	Time	<0.001
15 min	9.61 ± 7.27 †	11.81 ± 10.51 †	10.60 ± 11.72 †	Group × Time	0.174
30 min	5.99 ± 4.86 †	7.94 ± 6.43 †	8.93 ± 14.03 †		
60 min	2.73 ± 3.01	3.04 ± 2.42 †	3.99 ± 6.62 †		

† = Different than 0 min (*p* ≤ 0.05).

## Data Availability

The data that support the findings of this study are available by reasonable request from the author upon permission from the sponsor (NNB Nutrition).
